# Secondary Mixed Infection in Typhoid Fever

**Published:** 1888-08

**Authors:** Bayard Holmes

**Affiliations:** 125 State Street


### The Medical Journal and Examiner.

VOLUME LVII. AUGUST, 1888. NUMBER 2.

### ORIGINAL COMMUNICATIONS.

### SECONDARY MIXED INFECTION IN TYPHOID FEVER.

BY BAYARD HOLMES, M. D.

Typhoid fever is a term applied heretofore to a complexus of symptoms, well recognized and defined, and accompanied by a peculiar lesion in the intestinal tract; but now to the invasion of a non-pyogenic micro-organism, however that invasion take place—whether by way of the intestinal or of the respiratory* tract, and whether or not accompanied by the classical symptoms which characterize abdominal typhus.

In this strict sense, typhoid fever is the infection of the typhoid bacillus, and its direct consequences. Any symptoms and results which are due to other micro-organisms are not parts of the typhoid disease, and must be looked upon as accidental complications. The rôle of the typhoid bacillus is now demonstrated beyond the possibility of a doubt. The bacilli are found in the greatest numbers in the infiltrated Peyer's glands, before ulceration begins. Thence they are carried to the mesenteric glands, which imperfectly act as filters and detain them a short time. In a little while the pulmonary circulation is invaded through the thoracic duct, and a pneumonia follows, which is one of the early symptoms observed in most cases of typhoid. Examination by means of sections of the lung at this time, shows capillary emboli containing the bacillus. Only a part of the microbes are retained in the capillaries of the lungs. Many, if not most of them, escape into the arterial circulation, and they are found in every organ of the body, giving rise, at times, to all the consequences that capillary embolisms entail. The spleen is enlarged, and cultures from its blood demonstrate the presence of the bacillus. Sections from the gland show small capillary emboli containing the non-vegetating microbe. The same conditions are found in the kidneys; and when the lesion is sufficient to allow the passage of albumen into the urine, the bacilli may be demonstrated either microscopically or by cultures.

An earlier invasion of the blood sometimes, if not always, takes place through the portal circulation. From the infected glands in the intestine, the bacilli force themselves through the wall of a neighboring vein, and are carried into the liver, where they are found in masses in the portal capillaries. (*Liebermeister*, pp. 105, 106).

The typhoid bacillus does not produce suppuration, it does not completely destroy living tissues, except when in masses sufficient to produce large infarctions as in the spleen and kidneys. It does produce a toxæmia or sepræmia, and later, a septicæmia, which has a tendency to self-limitation. In the light of bacteriology it seems of itself to be far less fatal than the statistics would indicate. A careful consideration of the

* Klebs, Algemeine Pathologie, June, 1887, I, 165-175.

various and grave consequences which follow this disease, leads us to think that most of them are due to secondary invasion with bacteria which have nothing to do with the disease itself, and any of which may be absent in a typical course of typhoid fever. Although the typhoid bacillus is found in the dead tissues which result from constant pressure and consequent arrest of circulation under the bony prominences of the patient, and though they vegetate and multiply there, there is no evidence that they produce any further destruction of tissue.

It is not, then, the work of the typhoid bacillus which we wish at this time to consider. Wherever the invasion of this bacillus takes place, whether in the superficial lymph-glands of the intestinal or of the respiratory tract, the inflammation in these glands due to the irritation of the bacillus and its ptomaine so diminishes their resistance that a secondary invasion with pyogenic and other bacteria is a very easy thing. Many of the pathogenic bacteria are only facultative parasites of man, living for the most part as messmates with him on the contents of his intestines, or, as some think, being necessary even to mammalian digestion. When the intestine or any part of it is dead, or the barriers which ages of association have thrown up are torn down by truamatism,or otherwise, the before harmless or even helpful bacteria set up a destructive, saprophitic colonization of the tissues of their host, and, in the neighboring living tissues they may produce suppuration, coagulation necrosis, hæmorrhagic infiltration, lymphatic engorgement, or any of the results which are so frequently demonstrated in the infectious diseases, dependent, of course, on the peculiar anatomy and physiology of the invading parasite. Of all the bacteria capable of becoming pathogenic, the pus-microbes are the most ubiquitous, and their influence is most disastrous to life. It will be well, then, to consider at some length the manner of infection with these parasites, alone, and then, afterward, some of the other kinds of microbic invasion separately.

In addition to the local lesion in the intestine or larynx, the facility of infection with the pus-microbe is increased by the general condition of the patient brought about by the simple typhoid disease. The nutrition of the tissues is reduced to a minimum, the circulation is impeded directly by numerous capillary emboli, and indirectly by a diminished nutrition of the heart muscles. The poor quality of the blood and the retarded circulation invite the formation of thrombus. The lymphatic circulation is equally impaired. While under ordinary circumstances of health, the lymph apparatus has not only a great power of resistance to bacterial invasion, but also a remarkable power of destroying the invader, a few days of typhoid infection is enough to interfere with this function materially.

The infected and engorged Peyer's gland is very soon attacked by passing suppurative bacteria. Ulceration and sloughing follow in proportion to the destruction of tissue by the second infection. At this time, the symptoms begin to show that a new factor has begun to operate. The temperature is less regular in its remissions, and takes on a septic character similar to surgical-wound diseases. The micrococci are carried on into the mesenteric glands, where they may, in favorable cases, be arrested and destroyed. The glands after being enlarged for a long time return to nearly the normal size. The fatty degenerated material is removed, and the residue becomes calcified.

Unfortunately, this happy issue is not always realized. The filtering power of the already over-taxed gland is overcome, and the great lymphatic channel is flooded with the escaping bacteria. They are poured into the venous circulation, and find their way directly into the lungs. Here capillary embolism results a second time, and with the presence of a parasite which is capable of producing a destructive inflammation. This is the pneumonia which Murchison says "rarely appears before the third or fourth week," and then "may terminate in small abscesses, or, rarely, in gangrene" (p. 557). It must not be supposed that the presence of the pus-microbe is the only essential to the formation of destructive inflammation, or that, even in tissues the vitality of which is so much reduced by disease as the lungs in the third week of typhoid, they would invariably set up the suppurative process. The investigations of DeBary and Grawitz lead us to think that the resistance of the tissues is a much more important and powerful factor than we had supposed. But not all, if even a small part, of the bacteria are arrested in the capillaries of the lungs. Many of the emboli here are, no doubt, taken up by the pulmonary lymphatics and carried to the mediastinal glands, to be destroyed. Enlargement of these glands is frequent, and their breaking down into abscess is occasionally noticed.

Upon the arterial side of the circulation, the resistance of the tissues is, upon the whole, better preserved; but infection of bones, joints, and other serous cavities, and of the large organs of the body does take place. Then all the severe symptoms of osteomyelitis, suppurating synovitis, pericarditis, pleurisy, peritonitis, meningitis, and abscess in the large organs are added to the typhoid history. It is no wonder that the patient, already reduced by weeks of disease, is unable to resist this unexpected invasion, and very soon succumbs. These complications make up a very considerable bulk of the fatalities from abdominal typhus, though each in itself is rarely met with.

There are cases in which it seems that the bacteria of suppuration circulate in the blood freely until they are arrested by the arterial capillaries, and are then removed by the lymphatic apparatus. It is only in this way that we can account for the disappearance of bacteria experimentally injected into the arterial side of the circulation. For a time they are found in the blood, but in a few days no trace of them can be found by means of cultures. Sometimes, however, when there has been nothing to indicate that the tributary parts have been occupied by infection, the centrally located lymphatic glands swell up, and even undergo a destructive inflammation from the arrested pathogenic microbes. In the sequelæ of typhoid, adenitis is noticed by all systematic authors. By some it has been considered a favorable symptom (Chomel), while others have scarcely ever seen a case recover in which it had occurred. Of all the packets of glands it seems that those of the axilla are most frequently the seat of this form of secondary suppuration. One case is reported in which suppuration, beginning in the axilla, extended to the elbow, and into the thorax, and produced death by perforating the axillary vein. The patient died of uncontrollable venous hæmorrhage. In a case which came under my own observation this secondary infection of the axillary glands produced symptoms which resembled a relapse in the typhoid.

"Miss A., age 20, had been ten days convalescent from a very severe typhoid. Her temperature had been high, requiring the vigorous use of antipyretics. Upon the thirty-ninth day of her sickness, and the fourth day on which her evening temperature was normal, she began to eat solid food. Two days later the evening temperature went up to 100° Fahr. and continued to rise about a degree higher every night, until on the forty-seventh day of the sickness it was 103°. The physician who had attended her being taken sick at this time, I was called to the case. I found the patient very much emaciated. The abdomen was not tender or tympanitic. The stools were well digested and solid. The tongue was not dry and black, but lightly coated. There was ravenous hunger, and dreams of food disturbed sleep. The patient had been put back on a milk diet as soon as the temperature had declared itself. There was a poultice in the right axilla. The glands of the axilla were severally enlarged to an inch or more in diameter. One of the more superficial ones was discharging a small amount of pus through a follicle of the skin.

"Thinking that the condition of the axilla was a sufficient cause for the return of the temperature, I restored the more nutritious diet, removed the poultice and had the axilla shaved and covered with a wet carbolized dressing. On the following day, with the help of Dr. H. H. Frothingham, I anæsthetized the patient with chloroform and extirpated the glands of the axilla. The result is shown in the chart which I present. It was kept by the nurse. Although I failed to secure a perfectly aseptic wound, the temperature fell in four days to the normal, and the patient made an uninterrupted recovery."

Thus far we have considered the infection of a typhoid with the pus-microbe, the balance of power being, upon the whole, all the time on the side of the host. This condition of affairs can not always be maintained. The tissues of the body are all more and more impaired in the continuance of the disease, and, at last, a time comes when the invasion of the coccus is unresisted. Abscess formation takes place in all parts of the body. These foci may not reach a great size before death terminates their progress. This is especially the case in the abscesses of the brain, which Popoff has shown are almost always miliary and multiples. But besides the multiplication of the bacteria in definite localities, it may take place in the circulating blood itself. A small thrombus forms about the microbe, or a phragocite fails to devour its prey, and itself becomes food for the conqueror. In these ways the bacteria vegetate and multiply in the blood and lymph. The patient is in a state clinically termed septicæmia. This is, perhaps, the condition into which all cases of pyæmia ultimately verge.

But the disasters which the pus-microbe may precipitate upon the typhoid are not yet all rehearsed. At the first point of attack the destruction may be so great as to involve all the coats of the intestine, and perforation take place. In this way the largest serous cavity of the body is at once hopelessly infected, and fatal issue is not long delayed. Sometimes adhesions take place in advance of the destruction, and the perforation may be into a connective-tissue space about the cæcum, or into one of the large organs, or into another intestine. When the destruction involves a large blood-vessel in which an anticipating adhesive inflammation has not taken place, hæmorrhage may result, which, at once, or after many repetitions, may lead to dissolution. There is reason to think, however, that this is not the only cause of intestinal hæmorrhage in typhoid. When the eroded vessel is a vein, the septic bacteria may be carried into the portal circulation, and set up suppuration in the liver. In this way abscess of the liver may arise rather early in the disease, and before general pyæmia appears.

When the low state of nutrition of all tissues has reduced the resisting power to a minimum, infection may take place in any part of the body where germs are present. It is in this way that old scars open, from the germination of lasting spores, and old bone-disease lights up again. The secreting glands of the body may be invaded by way of their ducts. Suppuration of the parotid is not infrequent, and some cases of suppuration of the gall-apparatus without abscess of the liver are explainable only in this way.

Through the Eustachian tube the middle ear is invaded, and extension may go on into the brain, resulting in abscess or meningitis. In the same manner the sexual tract may offer a way to invasion, and orchitis, ovaritis and peritonitis supervene. Slight traumatisms result in defects of the skin, which immediately become the seat of protracted suppuration or other forms of infection.

It is probable that through the constant picking a way is made for infection with erysipelas, which so frequently appears about the nose and lips of typhoids. Tetanus also may make its way through the abraded skin or intestinal mucous membrane.

That a specific infection is the cause of the disease which Ceci terms hæmorrhagic infiltration, is now well demonstrated. This is the secondary infection which causes the uncontrollable epistaxis in diphtheria. There can be no doubt that the same form of infection is responsible not only for the epistaxis of typhoid, but, also, for a great many cases of diffuse intestinal hæmorrhages and hæmorrhages from the stomach and colon.

Noma, malignant œdema, and diphtheria present no anomalies in their appearance in typhoid.

There is a form of infection to which the poor typhoid is exposed which is the most pitiable of all. Either from the presence of the lasting spores of the bacillus, or from infection through the milk and other food, or through the inspired air, tuberculosis is a very frequent sequela of typhoid. All systematic writers notice this frequency, and attribute it to the protracted depression of the disease. Murchison says that it is more common after typhoid than after typhus, and that it is to be feared in all cases when hectic fever and bronchitis persist after the end of the fourth week (p. 558). Louis records four cases of section in which the lungs were found studded with recent tubercle. The frequency of tuberculosis and typhoid in Ireland has led Kennedy to suspect some essential relation between them. There is no doubt that the low state of vitality to which the patient is usually reduced and the slow convalescence give the latent spores in old glandular foci or in the cicatrices of the lungs an opportunity to vegetate again, and at the same time offer easy access to more virulent bacilli from tubercular nurses, or patients, or food.

It is to be regretted that no extensive and systematic study of the complications of typhoid have been clinically made in the light of modern bacteriological research. The use of cultures from aspirated products in the course of typhoid has been only just begun. Such researches carried on diligently for a few years would throw great light on many dark corners of symptomatology and pathology. So far we can only say that the work done allows us to reach theoretically and provisionally the following practical conclusions:

The local effects of an invasion with the typhoid bacillus is a non-destructive one, and the tendency is toward complete restitution to a state of health.

The primary lesion in the bowel or in the larynx gives rise to a point of least resistance; and the general impairment of nutrition renders all those causes which ordinarily determine the localization of infection far more potent.

Pyogenic and other forms of infection do take place through the primary lesion, and result in more than ordinarily serious consequences on account of the diminished resistance of all the tissues of the body.

Therefore all traumatism to the abdomen, either external, through violent, carless, or unnecessary palpation, or internal, through the use of food containing solid particles which might cause abrasion, should be strenuously avoided.

The imminent danger of typhoids to tuberculosis is conceded by all, and every precaution should be taken to prevent infection through contact with phthisical patients or nurses, or through confinement in rooms occupied by them, or through utensils or food which might furnish the infection; and when there is reason to suspect latent tuberculosis, the use of all anti-tubercular measures is recommended.

The treatment of typhoids and phthisical patients in the same hospital ward is little short of criminal, and the employment of tubercular nurses, attendants, or cooks, or ward-servants is incompatible with the present state of our knowledge of tubercular ætiology.

As typhoids are more than ordinarily susceptible to all contagious diseases, they should be rigorously excluded from direct and indirect contact with diphtheria, erysipelas, and all wound diseases; the most thorough cleanliness should be observed about their person, and the towels, bedding, and utensils should be beyond reproach.

In the care of the lips, the tongue, and the nose, care should be taken that no abrasions be made which might open a way to secondary invasion.

So-called relapses are often due to a secondary mixed infection. Therefore, in all cases of relapse, careful, diligent, and, if necessary, repeated search should be made for foci or infection which could give rise to the symptoms of relapse or any anomaly of temperature.

When a localization of infection has been discovered, the fact that the patient is, or has been, suffering from typhoid does not interdict the employment of ordinary surgical principles, but furnishes an additional and imperative indication for speedy operative interference, as furnishing the only known means of preventing the most disastrous issue.

NOTES AND LITERATURE.

It is unnecessary to refer to the literature of typhoid from a bacterial standpoint. The battle between mysticism and materialism was fought on the field of Anthrax and Tuberculosis before the real study of typhoid began. The difficulties in staining and in obtaining pure cultures of this microbe retarded its advance into the place it now occupies as the prime ætiological factor of abdominal typhus. Every modern text book on medicine or pathology gives a sufficient history and bibliography of this phase of the disease. The last German edition of Ziegler's Pathology, Klebs' Algemeine Pathologie, and Baumgarten's Lehrbuch der Pathologischen Mykologie are especially recommended.

Gottstein says that the complications of typhoid are not caused by the typhoid bacillus, but by the various pus-microbes. The importance of these complications may be inferred from the space devoted to their consideration by the systematic writers and in the current literature. More than ten quarto pages are devoted to the articles on this subject alone in the Catalogue of the Library of the Surgeon-General's Office. No statistics are available to show the fatality of the complications as compared with the pure disease. Baumgarten (l. c., p. 526) makes the remarkable statement that, under proper treatment, ninety-five or more per centum of typhoids recover.

The fact that the typhoid bacillus does not produce suppuration or other destructive lesion is so well established by numerous observers that the anomalous observation of an encysted suppurative peritonitis by Frankel (Centralb. f. Bact. u. Parask., 1887, I., p. 546) must wait further explanation. He found only the typhoid bacillus in the pus from this peritonitis, which occurred long after convalescence from a typhoid had been established. Baumgarten thinks the pyogenic bacteria had died at the time of the observation (p. 524). Seitz (Bacteriligische Studien sur Typhus Ætiologie, 1886) finds that this bacillus is not pyogenic in animals, although pathogenic in some. Mafucci (Centralb. f. Bact. u. Parastk., 1887, I., p. 149) concludes from experiments on animals that the bacteria are eliminated through the excretory and secretory glands without any destruction to their epithelium or the capillaries.

The most valuable information has been obtained from the following works:

Murchison: A Treatise on the Continued Fevers of Great Britain. Third edition. London. 1884.

Liebermeister: In the Cyclopedia of the Practice of Medicine, von Ziemsen's. American Translation. New York. 1874. Vol. I.

Seitz, Dr. Franz: Der Abdominaltyphus nach langjaehriger Beobachtung. Stuttgart. 1888.

Gottstein: Die Verwerthung der Bacterologie in der klinischen Diagnostik. Berlin. 1887.

DeBary and Grawitz: Ref. Centralb. f. Bact. u. Parastknd. 1887.

Dunin: Ueber die Ursache eiteriger Entzündungen und Venenthrombosen im Verlauf des Abdominaltyphus. Deut. Arch. f. klin. Med., XXXIX, pp. 369–392.

In an epidemic of typhoid in which complications were numerous, he made many examinations and cultures from the suppurative products, and found, in all cases, the pyogenic microbe. While his method leaves much to be desired, it is a very complete demonstration of the true cause of these accidents which had been previously only conjectured.

Metschinkoff: Phragocitic microbes in the relapses of Typhoid Fever. Virchow's Archive, Vol. 105–109, 1887–88.

W. H. Thompson: Diphteritic Paralysis. *Medical News*, June 9, 1888, p. 630.

CONDITION OF THE MESENTERIC GLANDS.—Before any secondary infection has taken place, they are enlarged and filled with the typhoid-bacillus, which is never found within the cell elements (Klebs, Baumgarten, and Liebermeister). In a few cases post-mortem examination has discovered the suppuration of these glands resulting in large abscesses, but there is abundant evidence that perforation does frequently take place in an intestine and recovery follow. Such is probably the explanation of the case of a late interne at the Cook County Hospital, who began to suffer a relapse two or three months after typhoid. Besides the peculiar renal and cerebral symptoms which he suffered, his temperature was not characteristic of typhoid, and, at last, a considerable amount of pus was discharged from the bowels. This was occasionally repeated after exacerbation of the symptoms throughout the course of a year or more.

Jenner (*Medical Times*, Vol. XX) records a case where perforation into the peritoneum followed suppuration of a mesenteric gland.

Andral (*Clinique Medical*, Paris, 1834, I, p. 599) observed the correspondence between the degree of ulceration in the intestinal (Peyer's) lymphatics and the engorgement of the mesenteric glands, and says that MM. Petit and Serres have compared this engorgement to that which follows in the crural and axillary glands in infection in the area tributary to them.

THE LUNGS.—Although the infection of the typhoid bacillus produces a pneumonia, it invariably tends to speedy recovery, and is observed only early in the disease. When, however, the secondary infection appears, the symptoms are of a much graver character, depending on the destructive power of the pyogenic infection.

Liebermeister (p. 172) calls attention to the fact that severe and fatal embolic pneumonia occurred in Bâsle when the hospital was overcrowded and in bad condition. This symptom disappeared when these defects were corrected.

This secondary pneumonia is ætiologically a unit, whether it appears as abscess, or gangrene, or suppurative bronchitis, and each may or may not be accompanied by suppurative pleuritis.

As we should expect, this disease appears about the third or fourth week. The lungs and the pleural cavity may become infected with putrid and gas-forming bacteria through the inspired air. (See a case by Witkowski, Virch. and Hirsch, *Jahresbericht*, 1872, II, p. 239, and Delafield, *N. Y. Medical Record*, 18—(?)

Pleurisy is not infrequent (Liebermeister, p. 173). It was observed in sixty-four out of 1,743 cases at Bâsle, of which twenty-one came to section.

INFECTION OF GLANDS BY WAY OF THEIR DUCTS.—All authors mention the frequent occurrence of parotitis, often in the course of a general pyæmia, but more often as the only evidence of infection. Careful study of parotitis, as it occurs in other conditions, leads me to think that the infection by way of the duct is not the rule. Stephen Paget (*Lancet*, 1886) attempts to establish some relation between the pelvic organs and the parotid gland. He remarks that parotitis is frequently the only secondary lesion to injury or operation upon the pelvis. He has collected sixty cases, including ten of Goodall's, only a few of which manifested any symptoms of pyæmia. I am inclined to think that the determination of infection in the parotid gland will be found to depend on some histological or physiological peculiarity of the gland, inviting the formation of thrombi.

Suppuration of the gall-apparatus is certainly occasionally noticed post-mortem, but oftener symptoms referable to inflammation of the common duct are recognized during life. Both of these conditions are dependent on the invasion of some form of bacteria, either alone or assisted by protozoa, during the depression of the typhoid disease.

In a few instances the pancreas has been found inflamed. In no case do I find that suppuration has been actually observed. There does not seem to be any reason why it should not occur (Andral).

Other small glands may become infected in the same manner. The middle ear thus becomes the seat of suppuration through the invasion, by way of the Eustachian tube (Seitz, 85).

This inflammation may invade the temporal bone, and suppurative meningitis or abscess of the brain result (Murchison, p. 561).

Joints and Other Serous Cavities.—A concise statement of the ætiology and clinical appearances of joint diseases in the course of convalescence of the acute infectious diseases, is given by Max Schueller (Die Pathologie und Therapie der Gelenkentzündungen, Wein u. Leipzig, 1887), with a full account of the literature. The large joints are most affected. Usually the invasion takes place late (19th to 60th day) in the disease. It is certainly rare, as in 3,130 cases in Vienna, Guttenbock found only two complicated by joint disease (Arch. f. klin. Chir., XVI, p. 61). Barwell (Diseases of the Joints, N. Y., 1881, p. 79) says that he has observed a large number of cases of this rare disease. He notices a painless mono-articular variety, and a painful poly-articular variety. If this is really the case, it does not seem difficult to account for the difference. The former is probably a tubercular or attenuated pyogenic infection, while the latter is a more destructive and virulent form. Murchison considers joint affection a sign of a fatal termination of the typhoid disease. Langenbeck (Akiurgie, p. 155) says that suppuration of the knee-joint as a complication of typhoid is an indication for resection, and Hager (Deut. Zeitschr. f. Chir., XXVII, p. 155) mentions two cases treated by antiseptic irrigation of the joint.

The bursæ in various parts of the body are only rarely mentioned as the seat of secondary invasion. I can find no case where it was not a part of a general pyæmia. There is no doubt that it does occasionally occur separately, but is then not considered of enough account to require mention.

Pleurisy, pericarditis, and peritonitis are more frequently mentioned than meningitis, but no one of them is often found as the only locus of infection.

Bones and the Large Organs of the body.—Cases of bone disease originating in the course of typhoid are mentioned by all authors. Murchison (p. 584) has observed in his own practice two cases of disease of the tibia, one of the femur, one of the temporal bone, and two of the lower jaw.

Old bone diseases frequently break out during typhoid, and scars that have been long healed open spontaneously and suppurate (Liebermeister, p. 146). These phenomena may be due to the germination of lasting spores, or to the localization of infection in the non-resisting scar. The same thing occurs in the pelvic cellular tissue (Zeigler, l. c., II, p. 894).

Abscess of the liver is a very common and fatal complication of typhoid (all authors). Two forms are to be considered: One, the result of infection through the portal circulation, appears early (7th to 11th day) in the disease, and is followed by pyæmia, or. especially, embolic pneumonia, and the other, due to arterial capillary thrombosis or embolism, appears as late as the third or fourth week, or long after convalescence has been established. Bender (*Lancet*, Oct., 1874) and Burger (Arch. f. klin. Med., XII, p. 623) have recorded cases of the former, and many authors of the latter (Murchison, Liebermeister etc.)

Abcesses of the brain are not very rare, though they do not often attain any great size (Gowers, Diseases of the Nervous System, 1888, p. 1215). They are mostly miliary and very numerous (Popoff, Virch., Arch., Bd. 87). One of the first operations for abscess of the brain is reported by Heineke (Die chirurgisch. Krankheit des Kopfes, p. 99). It occurred after a typhoid, and is supposed to have ended in recovery.

Suppuration of the thyroid is of unusual frequency. It occurred six times in 1,700 cases of typhoid (Liebermeister, p. 174). This frequency can hardly be accounted for by the size of the gland, and must depend on some anatomical peculiarity.

Infection of the bronchial and mediastinal glands by way of the larynx is of rather rare occurrence, but of a very fatal character. It is accompanied by œdema of the glottis, and often extensive destruction of cartilage (Seitz, pp. 85, 86). Murchison (p. 558) says that it appears to be very common in Germany.

All authors agree that bubo is more frequent after typhoid in the axilla than in the groin or in other packets of glands. This can, perhaps, be accounted for by supposing infection of the fingers and hands to take place from the mouth by biting the nails and picking the lips and teeth. In the Library of the Surgeon-General's Office, are three monographs on Axillary Phlegmons.

HÆMORRHAGIC INFECTION (CECI).—In diphtheria the unfavorable significance of repeated hæmorrhages from the nose has long been recognized by all clinicians. It has been shown by Klebs (p. 199) to be due to a secondary specific infection which he asserts is often the cause of epistaxis in typhoid. The same form of infection has been noticed in other localities. Frequently an intercellular hæmorrhage has been observed in the new-born which has been attributed heretofore to the "hæmorrhagic diathesis." Ritter has demonstrated it to be due to hæmhorrhagic infection through the umbilical wound. That the intestinal hæmorrhages of typhoid are due to the same specific microbe has not, so far as I know, been proved by cultures. Seitz (Case 51, p. 185) gives an account of a case in which severe pain in the stomach in the third week was followed by repeated gastric hæmhorrhages. Murchison (p. 530) mentions a case in which intestinal hæmorhage took place from diffuse spongy patches of excrescences firmly attached to the intestinal wall. The mesenteric glands and the spleen were enlarged. When hæmorrhage occurs in many places besides the bowel, the typhoid has been clinically termed "hæmorrhagic putrid fever" (M., p. 527). Out of 107 cases of epistaxis mentioned by Liebermeister (p. 175), fourteen cases were accompanied by hæmorrhage from other parts of the body, especially from the bowels. It usually occurs in young people, who are known to suffer most frequently from hæmorrhagic infection in other diseases. The appearance of the hæmorrhagic infection is sometimes alarmingly rapid. Ruggein (Virch. & Hirsch's Jahresberischt, 1872, II, p. 237), records such a case. An eighteen-year-old man was suffering a rather mild course of typhoid, when in the second week he was prostrated by an intestinal hæmorrhage accompanied by hæmorrhages from every observable surface of the body. All the secretions and excretions were bloody. Nevertheless convalescense began in the fifth week.

GAS-FORMING BACILLI (MALIGNANT ŒDEMA).—Although emphysema is frequently the result of an ulceration and perforation in those parts of the respiratory apparatus from which the atmospheric pressure is removed in inspiration (Murchison, p. 557) there are cases in which a secondary infection with a gas-producing bacillus is perfectly demonstrated.

Such an infection was observed by Meigs (*Philadelphia Medical Times*, Oct. 5, 1872), in which the liver was emphysematous throughout before death, and the case came to post mortem on the fourteenth day.

DIPHTHERIA.—Murchison (pp. 558–588) mentions three cases by Louis, two by Forget, and six by Rilliet and Barthey, and one of his own observation. In this case diphtheria appeared on the thirtieth day, and a local infection of the pus-microbe produced a post-coracoid abscess. Other illustrations are not wanting in the literature.

NOMA.—This has been observed by most systematic writers as an extremely rare complication. Murchison has met with it once. Greissinger noticed only one in 600 cases of typhoid. Out of eighty-nine cases of noma observed by Tourdes seven followed typhoid fever. West observed two similar cases. (See Murchison and Liebermeister.)

ERYSIPELAS.—This usually appears in an advanced stage of the disease (Murchison, p. 582). It appears in about 1 per centum of all cases. Seitz has rarely seen erysipelas in the early weeks of typhoid, but often in the later weeks or in convalescence (page 104).

TETANUS.—One of the most frequent and fatal of mixed infections in wound diseases is tetanus, which is also occasionally a complication of typhoid. Seitz (Cases 11 and 12, pages 80–83) gives the history of two cases, the result of which indicates that it is not always fatal.

TUBERCULOSIS.—Murchison observes that tuberculosis of the lungs is more common after typhoid than after typhus, and that tubercle ought always to be feared when hectic fever and bronchitis persist after the fourth week. Louis records four cases of fatal typhoid in which the lungs were studded with recent tubercles, and Bartlett observed long ago that consumption is a frequent sequela of this disease. Trouseau (*Union Medicale*, 1859) records cases of tubercular meningitis after typhoid. Harley and Kennedy (Murchison, p. 462) have even considered typhoid and tuberculosis dependent upon the same ætiological factor.

125 STATE STREET.

